# Ultrasonic complexation with *Lycium barbarum* polysaccharide significantly enhances the aqueous solubility and bioavailability of curcumin

**DOI:** 10.1016/j.ultsonch.2025.107673

**Published:** 2025-11-09

**Authors:** Li-Qiang Zhao, Zhuo-Qiong Li, Yue-Fan Liu, Meng-Ting Jiang, Ya-Nan Liu, Xin-Lan Zhang, Yu-Jie Sun, Jia-Lun Duan, Chun-Jie Bao, Jin-Ao Duan

**Affiliations:** a Jiangsu Provincial Key Laboratory of Functional Substances in Traditional Chinese Medicine Formulae and Innovative Drug Discovery, National and Local Collaborative Engineering Center of Chinese Medicinal Resources Industrialization and Formulae Innovative Medicine, Jiangsu Provincial Key Laboratory of Functional Substances in Traditional Chinese Medicine Formulae and Innovative Drug Discovery, Nanjing University of Chinese Medicine, Nanjing 210023, China; b School of Medicine, Nanjing University of Chinese Medicine, Nanjing 210023, China

**Keywords:** Ultrasonic, *Lycium barbarum* polysaccharide, Curcumin, Aqueous solubility, Bioavailability

## Abstract

Curcumin is a natural bioactive compound with a wide range of established health benefits. However, its practical applications are severely limited due to extremely poor aqueous solubility, which directly leads to low bioavailability. While polysaccharides like *Lycium barbarum* polysaccharide (LBP) can partially improve curcumin solubility, their solubilization efficiency remains limited. To overcome this challenge, we implemented ultrasonication as an effective processing strategy to enhance LBP’s capacity to promote curcumin dissolution. Our findings show that ultrasound-induced cavitation and related physico-chemical effects markedly improve LBP’s solubilization performance. The ultrasonically-assisted curcumin-LBP complex (CL-U) was systematically optimized through response surface methodology (RSM), identifying ultrasonic power, duration, and temperature as critical parameters. Extensive characterization verified that ultrasonication is essential for producing spherical core–shell nanoparticles, achieving a 2.23-fold enhancement in drug loading efficiency along with superior colloidal stability. Additional evidence from FTIR spectroscopy and acid hydrolysis experiments confirmed that ultrasonication reinforces hydrogen bonding as the principal intermolecular interaction stabilizing the complex. Biologically, CL-U demonstrated rapid cellular uptake in 4T1 cells within one hour and showed substantially improved antioxidant performance in both DPPH and ABTS assays. These functional gains are directly linked to the ultrasound-mediated improvements in solubility, stability, and bioavailability. This research establishes ultrasonication as a crucial sonochemical approach for constructing advanced polysaccharide-based delivery systems, providing a viable pathway for curcumin utilization in functional foods and pharmaceutical products.

## 1 Introduction

Curcumin, a yellow polyphenolic compound derived from the rhizomes of *Zingiberaceae* plants such as *Curcuma longa*, *Curcuma zedoaria*, and *Brassica juncea*, is widely utilized as a natural colorant, preservative, and food additive due to its notable antioxidant properties [1,2]. It exhibits diverse biological activities, including antimicrobial, anti-inflammatory, antiviral, and antitumor effects, and has demonstrated protective benefits for organs such as the liver, nerves, and heart [3,4]. For instance, Lan et al. reported that curcumin application in food surface treatment effectively delays oxidation and maintains freshness [5]. Other studies have confirmed that curcumin exerts anti-inflammatory effects by inhibiting nuclear factor kappa-beta (NF-κB), thereby reducing the release of inflammatory cytokines [6]. However, the practical application of curcumin is significantly limited by its extremely poor aqueous solubility—less than 50 nmol/mL—which is far below the minimum effective concentrations required for its bioactive effects, such as antioxidant (≥800 nmol/mL) and pro-angiogenic (≥1000 nmol/mL) activities [7,8]. This low solubility, coupled with unsatisfactory oral bioavailability, greatly restricts its utilization in the food and pharmaceutical industries. Consequently, the development of effective strategies to enhance the solubility and bioavailability of curcumin has become a crucial research focus.

Current approaches to improve the aqueous solubility of curcumin include surfactant solubilization, cyclodextrin encapsulation, and liposomal loading [9,10]. While these methods have partially ameliorated its solubility, issues such as inadequate stability, safety concerns, complex preparation processes, and poor biocompatibility remain unresolved [11]. In recent years, the application of diverse biomacromolecules (including proteins, polysaccharide, protein–polyphenol complexes, and protein-polysaccharide conjugates) has emerged as a promising strategy for constructing efficient delivery systems [[12], [13], [14]]. For instance, β-lactoglobulin–hyaluronic acid–neochlorogenic acid ternary complexes have been shown to significantly enhance the solubility of curcumin [15]. For instance, polysaccharides such as starch and chitosan have been employed to enhance the aqueous solubility of curcumin [16,17]. Overall, these macromolecular approaches demonstrate considerable potential in improving the solubility and stability of hydrophobic bioactive compounds, often through synergistic effects that enhance their overall functional performance. Among natural polysaccharides, *Lycium barbarum* polysaccharide (LBP)—a primary bioactive component derived from the medicinal and edible plant *Lycium barbarum*—has been widely investigated [18]. Studies indicate that LBP is mainly composed of monosaccharides such as arabinose, galactose, galacturonic acid, glucuronic acid, glucose, rhamnose, and mannose, featuring a complex spatial architecture and an abundance of exposed hydrophilic groups (e.g., hydroxyl and carboxyl groups), which contribute to its pronounced hydrating capacity and solubilization potential [19]. However, their efficacy in the native state is often suboptimal without additional physical processing.

However, studies have shown that the efficacy of polysaccharides such as starch in enhancing the solubility of curcumin in their native state is often unsatisfactory and typically requires additional physical processing to augment their solubilization capacity [17]. Ultrasonication has emerged as a highly efficient processing technology in the field of bioactive compound delivery [20]. The fundamental mechanism relies on acoustic cavitation, where the formation, growth, and violent collapse of micro-bubbles in a liquid medium generate extreme local temperatures, pressures, and high-speed micro-jets [21]. These intense physical forces can effectively disrupt molecular aggregates, reduce particle size, and enhance the interaction between hydrophobic compounds and hydrophilic carriers [22]. Compared to conventional methods, ultrasonication offers advantages such as rapid processing, controllability, and avoidance of organic solvents, making it particularly suitable for fabricating stable nano-delivery systems for poorly soluble bioactives.

Based on this background, the present study proposes the application of LBP as a natural solubilizing agent for curcumin, with the incorporation of ultrasonic treatment to further enhance the efficiency of the complexation process. Ultrasonication is expected to significantly improve molecular dispersion and interaction through cavitation effects, thereby optimizing the formation of LBP-curcumin complexes and more effectively increasing the dispersibility and solubility of curcumin in aqueous environments [23]. This study aims to systematically optimize the ultrasonication process, characterize the resulting complexes, and evaluate their potential for improving the delivery of curcumin.

## 2 Materials and methods

### 2.1 Materials

Curcumin (CFN98686, ≥98 %) was purchased from Tianzhi (China). Acetonitrile (6AS1122-001) was purchased from TEDIA (USA). 4T1 cells (CL-0007) was purchased from Procell (China). DPPH Free Radical Scavenging Capacity Assay Kit (A153-1–1) was purchased from Jiancheng (China). Total Antioxidant Capacity Assay Kit with a Rapid ABTS method (S0119) was purchased from Beyotime (China). Mannose (Man), Rhamnose (Rha), Arabinose (Ara), Galactose (Gal), Galacturonic acid (GalA), Glucose (Glu) and Glucuronic acid (GluA) were purchased from MedChemExpress (USA).

### 2.2 The extraction and identification of LBP

*Fructus Lycii* (Ningxia 3) were collected from the Ningxia region of China. The dried fruits were first extracted with 80 % ethanol at a solid-to-liquid ratio of 10:1 to remove lipophilic compounds. The resulting residue was dried and subsequently subjected to hot water reflux extraction using deionized water for one hour. The aqueous extract was concentrated under reduced pressure, and crude polysaccharides were precipitated by adding ethanol to a final concentration of 80 %. The crude LBP was further deproteinized using the Sevage method (chloroform:n-butanol = 4: 1, v/v) repeatedly. The deproteinized fraction was then purified by anion-exchange chromatography on a DEAE-52 cellulose column, eluted with deionized water. The polysaccharide-rich fraction was collected, yielding refined LBP.

### 2.3 Analysis of monosaccharide composition

The monosaccharide composition of LBP was analyzed by HPLC following pre-column derivatization with 1-phenyl-3-methyl-5-pyrazolone (PMP). Monosaccharide standards, including Man, Rha, Ara, Gal, GalA, Glu and GluA, were used for qualification. The chromatographic separation was performed under the following conditions: an isocratic elution system consisting of acetonitrile and phosphate buffer (12 g/L potassium dihydrogen phosphate, adjusted to pH 6.8 with 2 M NaOH) at a volume ratio of 17:83. The flow rate was set at 0.8 mL/min, the column temperature was maintained at 30 °C, and the detection wavelength was 250 nm. The injection volume was 10 μL.

### 2.4 Molecular weight analysis

The molecular weight and homogeneity of the LBP were determined by High-Performance Gel Permeation Chromatography (HPGPC). The analysis was performed on a Waters 2695 high-performance liquid chromatography system (Shimadzu, USA) equipped with a Waters 2414 refractive index detector (RID). Separation was achieved using two gel filtration columns (TSK-gel G-3000 and 5000PW, 8.0 × 300 mm, 6 μm; Showa Denko Co., Tokyo, Japan) connected in series. The column temperature was maintained at 40.0 °C. A 0.1 M NaNO_3_ solution was used as the mobile phase at a flow rate of 0.8 mL/min. For the analysis, a calibration curve was established using a series of standard dextrans with known molecular weights of 2.70, 5.25, 9.75, 13.05, 23.00, 36.80, 50.00, and 67.00 kDa. The LBP sample was prepared by dissolving 2 mg in the mobile phase, followed by filtration through a 0.22 μm microporous membrane. An injection volume of 10 μL was used for the analysis. The molecular weight of LBP was calculated by comparing its retention time to the established calibration curve.

### 2.5 Single factor experiment

Curcumin (10 mg) was accurately weighed and dispersed in 60 mL of ultrapure water under vigorous shaking. The resulting suspension was divided into six equal groups, to which varying amounts of LBP (0.1, 1, 10, 15, 25, and 30 mg) were added, respectively. Each mixture was subjected to ultrasonication at 500 W and 25 °C for 30 min, followed by centrifugation at 4800 r/min for 10 min. The supernatant was collected, and its absorbance was measured at 430 nm. To investigate the effects of different processing parameters, the same procedure was repeated while systematically varying the ultrasonic power (100, 200, 300, 400, 500, and 600 W), ultrasonication time (10, 20, 30, 40, 50, and 60 min), and temperature (10, 20, 30, 40, 50, and 60 °C). The corresponding absorbance values were recorded for each condition.

### 2.6 Response surface optimization

The extraction conditions were optimized using response surface methodology (RSM), with the ultraviolet absorbance of curcumin at 430 nm serving as the response variable. The factors selected for optimization included ultrasonic power (A), LBP concentration (B), ultrasonic time (C), and ultrasonic temperature (D). Experimental design and analysis were conducted using Design-Expert 13 software based on a Box-Behnken design (BBD), with factor levels determined through preliminary experiments.

### 2.7 Infrared spectral analysis

Each sample (Curcumin, LBP, CL, CL-U) weighing 10 mg was mixed with potassium bromide (KBr) as a dispersing agent and compressed into a pellet. The pellets were then analyzed using a Fourier-transform infrared (FTIR) spectrometer (Nicolet Summit, Thermo Fisher Scientific, USA) by scanning over a wavenumber range of 4000 to 500 cm^−1^.

### 2.8 TEM examination

The sample (CL-U) was deposited onto a copper grid and negatively stained with uranyl acetate. Morphological characterization was conducted using a Tecnai G2 Spirit transmission electron microscope (Thermo Fisher Scientific, USA) operated at an acceleration voltage of 120 kV.

### 2.9 Quantification of curcumin

The quantification of curcumin in the samples was performed using ultraviolet (UV) absorbance measurement and HPLC. For the UV method, the absorbance of the samples was directly measured at 430 nm using a microplate reader. For HPLC analysis, chromatographic separation was carried out on an Agilent ZORBAX Eclipse XDB-C18 column (250 mm × 4.6 mm, 3.5 μm) maintained at 25 °C. An isocratic elution program was employed with a mobile phase consisting of 0.1 M phosphate solution (A) and acetonitrile (B) in a ratio of A:B = 1:1 (v/v) for 13 min. The flow rate was set at 1.0 mL/min, and UV detection was performed at a wavelength of 430 nm.

### 2.10 Zeta potential and particle size analysis

The hydrodynamic diameter, ζ-potential, and polydispersity index (PDI) of the samples were measured at room temperature using a laser diffraction particle size analyzer (Zetasizer Nano ZS90, Malvern Panalytical, UK). To evaluate the stability of the formulations, the hydrodynamic size and ζ-potential were monitored continuously over a period of four days.

### 2.11 DPPH radical scavenging activity

The DPPH radical scavenging activity was evaluated according to a previously reported method with slight modifications. 2 mL of the CL or CL-U complex at its preparation concentration (12.87 ± 1.96 mg/mL and 12.65 ± 1.32 mg/mL, respectively) was mixed with an equal volume (2 mL) of freshly prepared DPPH solution. The mixture was incubated in the dark at room temperature for 30 min. After incubation, the absorbance was measured at 517 nm by microplate reader. For each sample concentration tested, we prepared not only the test mixture (sample + DPPH solution) but also a dedicated “sample background control”. This control was prepared by dissolving pure curcumin in absolute ethanol at a concentration matching that present in the corresponding sample. This setup was designed to specifically quantify and subtract the absorbance solely attributable to the intrinsic color of curcumin at the measurement wavelength. The corrected formula used to calculate the actual DPPH radical scavenging activity was: Scavenging activity (%) = [ (A control − (A sample – A background)) / A control] × 100. Ascorbic acid and quercetin were used as positive controls in the experiment.

### 2.12 ABTS radical scavenging activity

The ABTS radical cation (ABTS∙+) working solution was prepared by mixing equal volumes of ABTS stock solution and potassium persulfate solution, followed by reaction in the dark at room temperature for 16 h. The resulting solution was then diluted with phosphate buffer until an absorbance of 0.70 ± 0.02 was achieved at 734 nm. For the assay, 2 mL of sample solutions at various concentrations (0.05–0.25 mg/mL) were combined with 2 mL of the diluted ABTS∙+ solution and incubated in the dark at room temperature for 30 min. The absorbance was measured at 734 nm after the incubation period by microplate reader. ABTS∙+ scavenging activity formula (%) = [1-(A sample − A control) / A blank] × 100.

### 2.13 Cellular uptake assay

4T1 triple-negative breast cancer cells were employed as the target cell line. The cells were treated with the sample-containing culture medium for 48 h. Following incubation, the cells were harvested and the fluorescence intensity was measured using flow cytometry to determine the cellular uptake efficiency of curcumin. In parallel, the cells were fixed and simultaneously stained with DAPI for nuclear visualization. Fluorescence microscopy was subsequently performed to observe the cellular localization of curcumin, with specific excitation/emission settings configured for detection at 478 nm.

### 2.14 Statistical analysis and reproducibility

Statistical analysis was performed with GraphPad Prism 7.0 (La Jolla, CA, USA). All values were presented as mean ± standard deviation (SD). Differences among multiple groups were assessed by one-way analysis of variance (ANOVA), while comparisons between two groups were carried out using an unpaired two-tailed Student’s *t*-test. A probability (p) value below 0.05 was deemed statistically significant. In figures, significance levels are denoted as follows: *p < 0.05, **p < 0.01, ***p < 0.001, and ns (not significant) for p ≥ 0.05.

## 3 Result and Discussion

### 3.1 Extraction and Identification of LBP

**Fig. 1:**
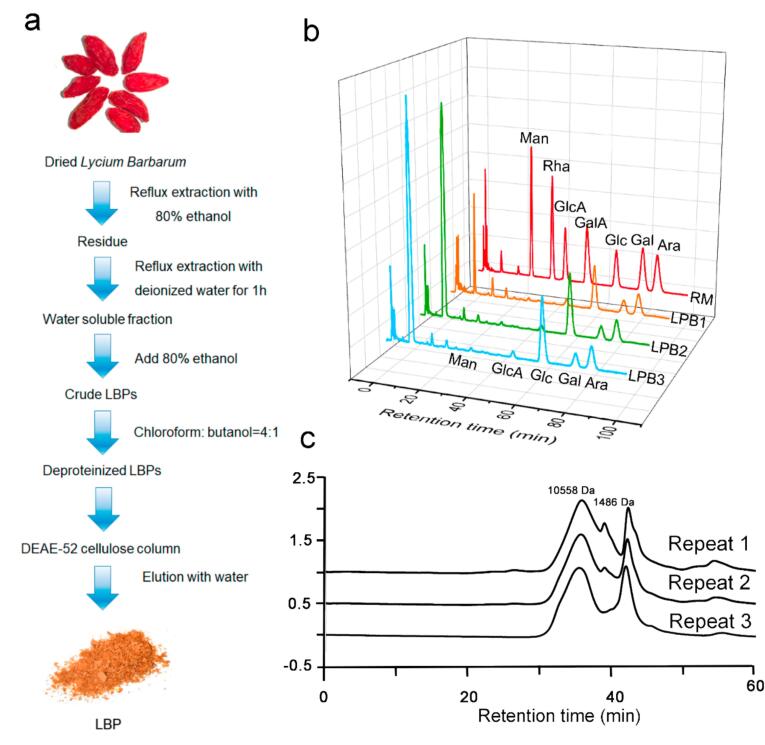
Extraction and Characterization of LBP. a. Extraction of LBP. b. Monosaccharide Composition Analysis of LBP. c. Molecular Weight Determination of LBP.

As Fig. 1a, LBP was extracted from the fruits of *Lycium barbarum* via hot water extraction, followed by ethanol precipitation to obtain crude polysaccharides. After deproteinization, the crude extract was further purified by DEAE-52 cellulose column chromatography. The resulting LBP exhibited a polysaccharide content of 70.82 ± 5.23 % as determined by the phenol–sulfuric acid method, and a protein content of 0.12 ± 0.11 % measured via the BCA assay. Monosaccharide composition analysis performed by PMP pre-column derivatization revealed that LBP consists primarily of arabinose, galactose, and glucose, with galactose being the most abundant monosaccharide at approximately 65.4 %. Furthermore, HPGPC indicated that the molecular weight of LBP predominantly ranged between 1000 and 11000 Da. These results collectively confirm the successful extraction and purification of LBP.

### 3.2 Analysis of Single-Factor experimental

#### 3.2.1 Effect of LBP concentration on the solubility of curcumin

**Fig. 2:**
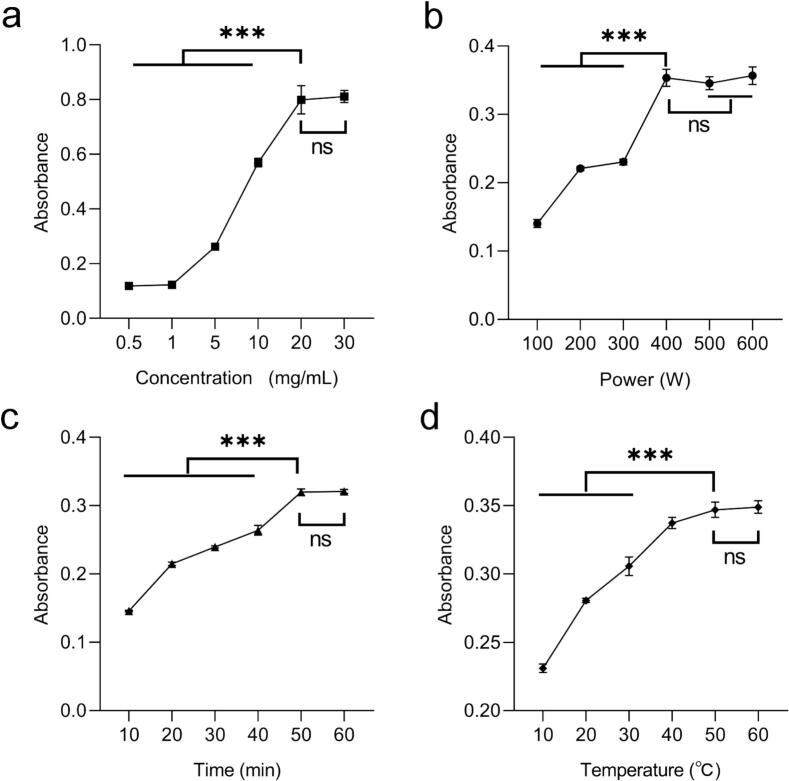
The influence of different extraction conditions on the yield of curcumin (a) Effect of LBP concentration on the yield of curcumin, (b) Effect of ultrasonic power on the yield of curcumin, (c) Effect of ultrasonic time on the yield of curcumin, (d) Effect of ultrasonic temperature on the yield of curcumin. Data are represented as mean ± s.d. ns, no significance, ***, p < 0.001n = 3.

To investigate the influence of LBP concentration on curcumin solubility, the dissolution behavior of curcumin was evaluated across a range of LBP solutions (0.5–20 mg/mL). As shown in Fig. 2a, the solubility of curcumin increased progressively with rising LBP concentration up to 20 mg/mL. This enhancement may be attributed to non-covalent interactions between LBP and curcumin, facilitated by the porous and flexible structure of the polysaccharide, which provides accessible binding sites and promotes molecular encapsulation. However, when the LBP concentration exceeded 20 mg/mL, no further improvement in solubility was observed, suggesting that the binding capacity between LBP and curcumin had reached saturation. Therefore, an LBP concentration range of 10–20 mg/mL is considered optimal for maximizing the solubilization of curcumin.

#### 3.2.2 Effect of ultrasonic power on the solubility of curcumin

As shown in Fig. 2b, the absorbance of curcumin in an aqueous LBP solution, measured using a microplate reader, increased with rising ultrasonic power, indicating enhanced solubility. This improvement is attributed to the vigorous mechanical agitation induced by ultrasonication, which promotes molecular interactions between LBP and curcumin. However, when the ultrasonic power exceeded 400 W, the solubility increase plateaued, with only marginal further enhancement. This saturation effect may be due to potential degradation of LBP under high ultrasonic intensity, where cleavage of glycosidic bonds and structural disruption of the polysaccharide could limit additional solubilization of curcumin [24,25]. Therefore, an ultrasonic power within the range of 400–600 W is considered optimal for achieving high curcumin solubility in the LBP aqueous solution.

#### 3.2.3 Effect of ultrasonication time on the solubility of curcumin

As illustrated in Fig. 2c, the solubility of curcumin in the aqueous LBP solution increased with prolonged ultrasonication time from 10 to 50 min. This suggests that extended exposure to ultrasonic energy enhances the interaction between LBP and curcumin, likely through improved molecular dispersion and complexation. However, further extension of ultrasonication beyond 50 min, up to 60 min, did not lead to a significant increase in solubility, indicating a saturation point in the solubilization process. Based on these observations, an ultrasonication time of 40–50 min is considered optimal for achieving efficient curcumin dissolution in the LBP solution.

#### 3.2.4 Effect of temperature on the solubility of curcumin

As depicted in Fig. 2d, the solubility of curcumin in the aqueous LBP solution increased progressively as the temperature rose from 10 to 40 °C. This enhancement may be attributed to the elevated kinetic energy of solvent molecules at higher temperatures, which facilitates molecular diffusion and disrupts the stable crystalline structure of curcumin, thereby accelerating the dissolution rate and increasing the saturation solubility. However, when the temperature was further increased to the range of 40–60 °C, no significant improvement in solubility was observed. This plateau suggests that the solubilization effect of LBP may have reached its maximum under these conditions, or that thermal degradation of curcumin began to offset the gains from increased temperature. Consequently, an optimal temperature range of 40–50 °C is recommended for achieving high curcumin solubility in LBP-based aqueous systems.

#### 3.2.5 Response surface experiment

Based on the single-factor experimental results, four factors exhibiting the most significant effects on curcumin solubility—ultrasonic power (A), LBP concentration (B), ultrasonication time (C), and ultrasonic temperature (D)—were selected for optimization using a Box-Behnken design (BBD). This approach was employed to systematically investigate the interactions among these variables and identify optimal conditions for enhancing the aqueous solubility of curcumin. The experimental design and corresponding responses are summarized in Table 1. A second-order polynomial regression model was fitted to the experimental data, yielding the following equation:Y = 0.8008 + 0.0865A + 0.2992B + 0.0950C + 0.0372D + 0.0187AB – 0.0400AC – 0.0127AD + 0.0265BC + 0.0002BD – 0.0270CD – 0.0834A^2^ – 0.2559B^2^ – 0.0749C^2^ – 0.0407D^2^. where represents the absorbance at 430 nm of curcumin, and A, B, C, and D correspond to ultrasonic power, LBP concentration, ultrasonication time, and temperature, respectively.

**Table 1:** Response surface experimental design and results.

Run	A: Power W	B: Concentration mg/mL	C: Time min	D: Temperature ℃	Absorbance
1	400	13	50	50	0.832
2	500	13	30	30	0.546
3	500	13	10	40	0.615
4	400	13	10	50	0.642
5	300	13	10	40	0.456
6	500	1	30	40	0.111
7	500	13	50	40	0.812
8	400	13	30	40	0.818
9	400	13	30	40	0.822
10	400	25	10	40	0.669
11	400	13	30	40	0.827
12	300	13	50	40	0.523
13	400	25	30	50	0.828
14	400	13	10	30	0.517
15	400	1	50	40	0.222
16	300	13	30	50	0.537
17	500	13	30	20	0.824
18	300	1	30	15	0.121
19	400	13	30	15	0.805
20	400	25	30	10	0.679
21	400	13	50	10	0.605
22	400	13	30	15	0.841
23	400	1	30	20	0.192
24	300	13	30	10	0.508
25	400	1	30	10	0.114
26	400	25	50	15	0.831
27	300	25	30	15	0.567
28	500	25	30	15	0.832
29	400	1	10	15	0.116

The analysis of variance (ANOVA) for the developed model is summarized in Table 2. The high coefficient of determination (R2 = 0.9958) and adjusted R2 (R2adj = 0.9916) indicate that the model explains a substantial proportion of the variability in the response. The model's significance is further supported by a high F-value of 237.05 with a very low p-value (p < 0.0001), demonstrating that the regression is statistically significant and fits the experimental data well. The non-significant lack-of-fit term (p > 0.05) confirms that the model adequately represents the observed data, with minimal experimental error. These results collectively validate the model’s reliability for predicting the response under varying experimental conditions.

**Table 2:** Analysis of variance (ANOVA)of the response surface quadratic mode.

Source	Sum of Squares	df	Mean Square	F-value	p-value	
**Model**	1.85	14	0.1320	237.05	< 0.0001	significant
A-Power	0.0881	1	0.0881	158.13	< 0.0001	
B-Concentration	1.04	1	1.04	1864.60	< 0.0001	
C-Time	0.0547	1	0.0547	98.18	< 0.0001	
D-Temperature	0.0654	1	0.0654	117.46	< 0.0001	
AB	0.0189	1	0.0189	33.95	< 0.0001	
AC	0.0042	1	0.0042	7.59	0.0155	
AD	0.0155	1	0.0155	27.83	0.0001	
BC	0.0008	1	0.0008	1.41	0.2552	
BD	0.0013	1	0.0013	2.26	0.1547	
CD	0.0026	1	0.0026	4.67	0.0485	
A^2^	0.1163	1	0.1163	208.91	< 0.0001	
B^2^	0.5092	1	0.5092	914.29	< 0.0001	
C^2^	0.0473	1	0.0473	85.00	< 0.0001	
D^2^	0.0496	1	0.0496	89.02	< 0.0001	
**Residual**	0.0078	14	0.0006			
Lack of Fit	0.0071	10	0.0007	4.13	0.0922	not significant
Pure Error	0.0007	4	0.0002			
**Cor Total**	1.86	28				

**Fig. 3:**
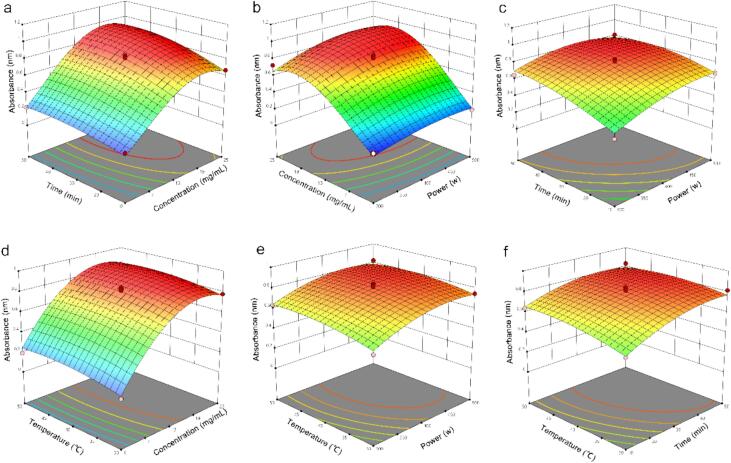
Response surface of two factor interactions on the on the aqueous solubility of curcumin. a. BC 3D surface map. b. AB 3D surface map. c. AC 3D surface map. d. BD 3D surface map. e. AD 3D surface map. f. CD 3D surface map.

Among the factors investigated, LBP concentration exhibited the most pronounced effect on the aqueous solubility of curcumin, followed by ultrasonication time and temperature, while ultrasonic power demonstrated the least influence. Interaction effects between factors were also evaluated. As summarized in Table 2 and illustrated in Fig. 3, the interactions between ultrasonic power and LBP concentration (A × B), ultrasonic power and time (A × C), ultrasonic power and temperature (A × D), as well as time and temperature (C × D), were statistically significant (p < 0.05). In contrast, the interactions between ultrasonication time and LBP concentration (B × C), and LBP concentration and temperature (B × D), did not show significant effects on curcumin solubility (p > 0.05).

To determine the optimal parameters for enhancing the solubility of curcumin, a regression model was employed to identify the following theoretical optimum conditions: ultrasonic power of 452 W, LBP concentration of 17.4 mg/mL, processing time of 44 min, and temperature of 43 °C. Under these conditions, the predicted absorbance value of the polysaccharide solution was 0.984. Experimental validation was carried out through triplicate parallel tests under the same parameters, yielding an average absorbance of 0.935 ± 0.32. The close agreement between the experimental and predicted values confirms the high predictive accuracy and reliability of the developed regression model.

### 3.3 Quantitative analysis of Curcumin-LBP complexes

The compositional profiles of curcumin-LBP complexes (CL-U) (Fig. S1), prepared under response surface-optimized conditions, were systematically evaluated using validated analytical methodologies. The polysaccharide content was determined via the phenol–sulfuric acid assay, while curcumin quantification was performed using HPLC. HPLC analysis demonstrated a substantial enhancement in curcumin incorporation following ultrasonication treatment (Fig. S2). The ultrasonicated complex (CL-U) exhibited a curcumin content of 2.058 ± 0.256 μg, representing a significant increase (p < 0.05) compared to the non-ultrasonicated control (CL, 0.924 ± 0.322 μg). This 2.23-fold improvement in curcumin loading efficiency strongly suggests that ultrasonication promotes molecular interactions between curcumin and the polysaccharide matrix, potentially through enhanced hydrogen bonding, hydrophobic interactions, and improved molecular dispersion. In contrast, polysaccharide quantification revealed no statistically significant difference (p > 0.05) between CL-U (12.65 ± 1.32 mg) and CL (12.87 ± 1.96 mg), indicating that the ultrasonication process does not lead to substantial polysaccharide degradation or loss. These findings collectively demonstrate that ultrasonication represents an efficient physical processing strategy that significantly enhances curcumin encapsulation in LBP complexes without compromising polysaccharide integrity.

### 3.4 Particle size and zeta potential analysis of CL-U

**Fig. 4:**
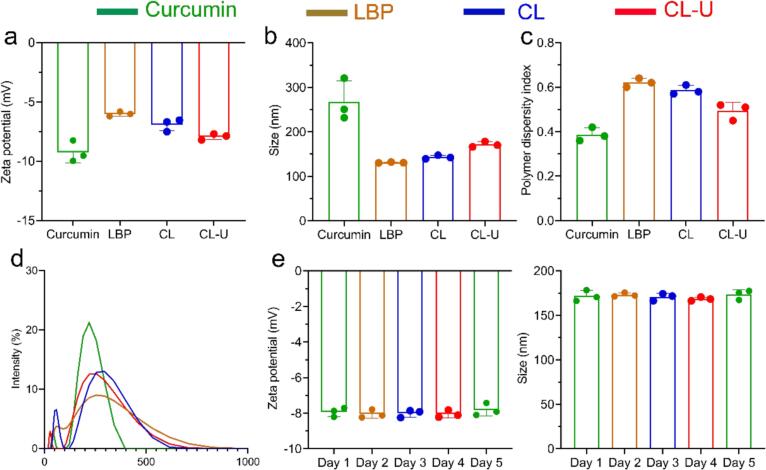
Zeta potentials, Size distribution and PDI of Curcumin, LBP, CL and CL-U. a. Zeta potentials. b. Size. c. PDI. d. Size distribution. e. Zeta potential and Size distribution of CL-U in five days.

Polysaccharides are recognized as ideal building blocks for nano-delivery systems due to their amphiphilic nature and flexible molecular chains [26]. These inherent properties enable them to effectively encapsulate hydrophobic bioactive compounds through self-assembly, thereby facilitating the formation of nanocomplexes [27]. Moreover, the intense mechanical effects and cavitation generated by ultrasonication can further optimize this assembly process. This strategy has been successfully employed in various polysaccharide systems, including alginate and starch, for the preparation of structurally uniform nanoparticles [[28], [29], [30]]. Based on the foregoing analysis, we hypothesize that ultrasonication promotes the formation of a stable nanostructure in the CL-U. To test this hypothesis and systematically evaluate the physicochemical properties of the complex, we selected particle size distribution and zeta potential as key indicators to assess nanocomplex formation efficiency and colloidal stability, respectively. As illustrated in Fig. 4, Fig. 4, both curcumin and LBP exhibited negative zeta potentials in aqueous dispersion, with values of −9.24 ± 0.73 mV and −6.00 ± 0.16 mV, respectively. These charges are attributed to the dissociation of phenolic hydroxyl groups in curcumin and the ionization of carboxyl groups on the LBP molecular chains. Notably, the zeta potential values for the CL (−6.91 ± 0.43 mV) and CL-U (, −7.92 ± 0.21 mV) were intermediate to those of the curcumin and LBP. This observation indicates that the complexes are not simple physical mixtures but likely form a “core–shell” structure, which partially shields the negative surface charge of curcumin. Notably, the zeta potential of CL-U was significantly more negative than that of CL and closer to that of pure curcumin. This trend is consistent with the content determination results, which revealed a substantially higher curcumin loading efficiency in CL-U. Collectively, these findings suggest that ultrasonication promotes a more thorough and compact encapsulation of curcumin by LBP.

Particle size analysis revealed that the hydrodynamic diameters of CL (143.30 ± 1.86 nm) and CL-U (171.73 ± 5.02 nm) fell between those of curcumin aggregates (281.80 ± 56.78 nm) and molecularly dispersed LBP (132.33 ± 1.86 nm) (Fig. 4, Fig. 4). This intermediary size distribution supports the formation of a “core–shell” nanostructure, wherein the hydrophilic LBP shell encapsulates the curcumin core, thereby sterically hindering further aggregation of curcumin. The larger particle size of CL-U compared to CL is likely due to its higher curcumin content. Moreover, the CL-U complex demonstrated excellent colloidal stability, as indicated by the negligible variations in particle size, zeta potential, and polydispersity index (PDI) over a five-day period (Fig. 4e).

### 3.5 Transmission electron microscope analysis of CL-U

**Fig. 5:**
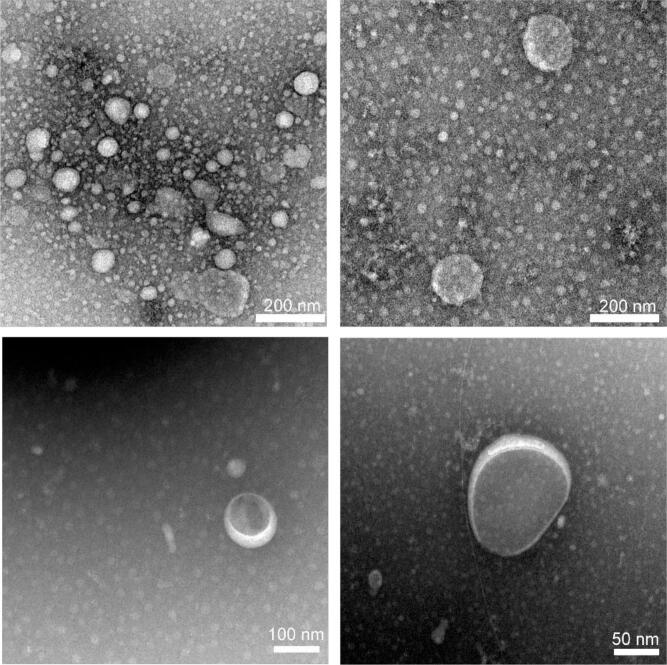
TEM of CL-U. a-b. 200 nm c. 100 nm d. 50 nm.

To further characterize the morphology of the CL-U nanocomplex, TEM was employed to observe its microstructure. As shown in the TEM (Fig. 5), the ultrasonically synthesized CL-U composite forms predominantly spherical nanoparticles. The particles display a clear contrast, with darker regions indicative of the electron-dense curcumin core, surrounded by a lighter, less dense shell presumed to be the LBP polysaccharide. The particle sizes observed under TEM are in general agreement with the DLS data (122 nm), although TEM measures the dry state while DLS measures the hydrodynamic diameter in solution. Furthermore, the well-dispersed nanostructure visualized by TEM provides direct morphological evidence that the remarkable enhancement in curcumin solubility is achieved through ultrasonication-driven nano-encapsulation, wherein the hydrophilic LBP shell effectively shields the hydrophobic curcumin core, rendering it readily dispersible in aqueous environments.

### 3.6 Infrared absorption spectroscopy analysis of CL-U

**Fig. 6:**
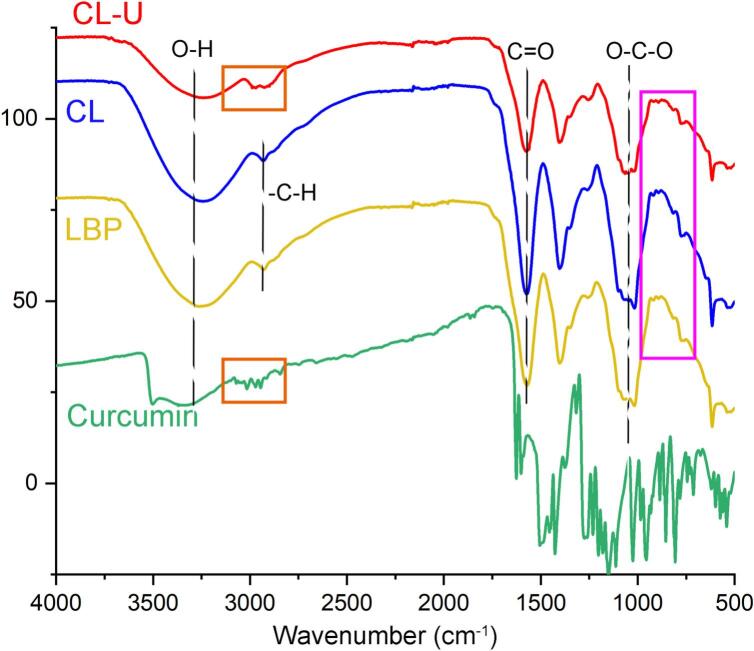
Infrared spectral analysis of Curcumin, LBP, CL and CL-U.

To elucidate the structural characteristics and intermolecular interactions within the complexes, FTIR spectroscopy was employed to compare curcumin, LBP, and their respective complexes (CL and CL-U) (Fig. 6). As shown in Fig. 6, all samples exhibited a broad O–H stretching vibration at approximately 3300 cm^−1^ and C–H stretching vibrations near 2890 cm^−1^. Characteristic absorption bands for curcumin were observed at 1752 cm^−1^, corresponding to C=O stretching, and in the 2800–3000 cm^−1^ region, attributed to –CH_2_ group vibrations. LBP displayed its signature absorption in the 700–1100 cm^−1^ range, consistent with C–O–C glycosidic bond stretching. A notable spectral change was the significant broadening of the O–H stretching band in CL-U at 3300 cm^−1^ compared to LBP, curcumin, and CL, indicative of enhanced intermolecular hydrogen bonding. This suggests that ultrasonication facilitates stronger hydrogen bond interactions between curcumin and LBP in the CL-U complex. Furthermore, the –CH_2_ stretching vibrations in the 2800–3000 cm^−1^ region were markedly more intense in CL-U than in CL, where they were scarcely detectable. This spectral evidence correlates with a higher curcumin encapsulation efficiency in CL-U, which is consistent with HPLC quantification results and confirms that ultrasonication significantly improves the loading capacity of the complex.

### 3.7 Cellular uptake efficiency analysis of CL-U

The poor aqueous solubility of curcumin is widely recognized as a major factor limiting its bioavailability [31]. The CL-U developed in this study was designed to address this issue by enhancing curcumin solubility. To evaluate whether this improvement in physicochemical properties translates into enhanced bioavailability, we investigated the cellular uptake behavior of the complexes in 4T1 tumor cells using flow cytometry and confocal laser scanning microscopy (CLSM).

**Fig. 7:**
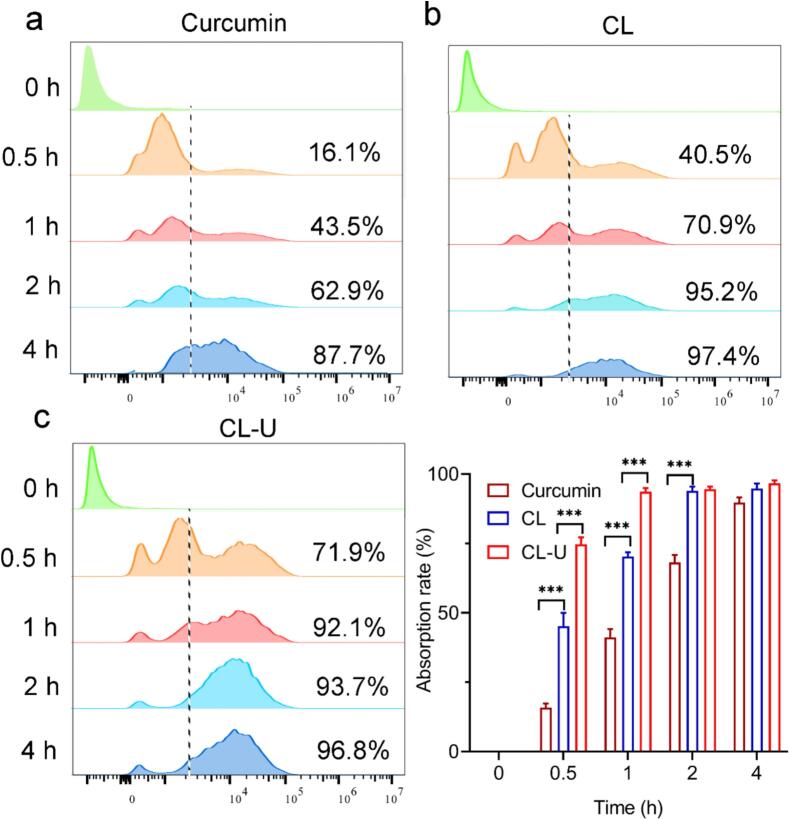
The cellular uptake of Curcumin (a) CL (b) CL-U (c) by 4T1 tumor cells was analyzed using flow cytometry.

**Fig. 8:**
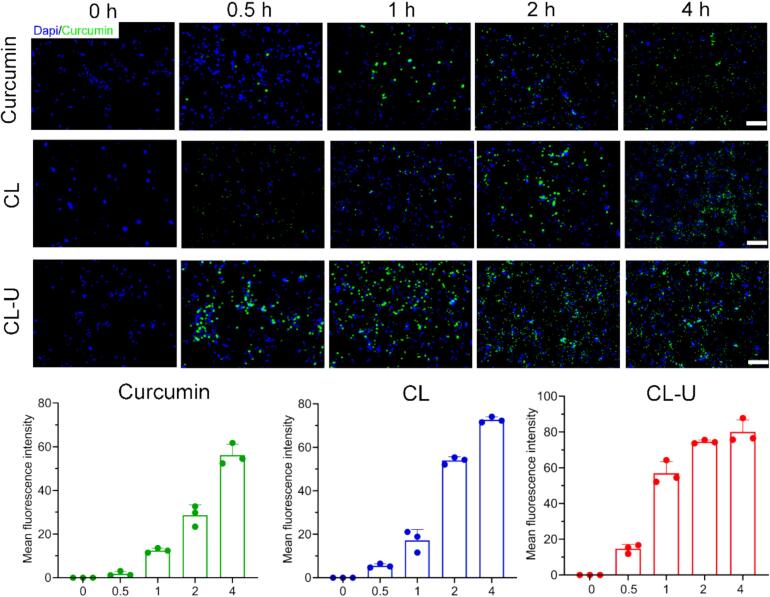
The cellular uptake of Curcumin, CL, CL-U by 4T1 tumor cells was analyzed using confocal laser scanning microscopy. Scale bars, 200 μm.

Flow cytometric analysis revealed substantially improved cellular internalization of the complexes compared to free curcumin. After 0.5 h of incubation, the cellular uptake rates were 16.1 % for free curcumin, 40.5 % for the CL complex, and 71.9 % for the CL-U complex. Notably, CL-U achieved complete cellular uptake within approximately 1 h, significantly faster than CL (2 h) and free curcumin (4 h) (Fig. 7). CLSM observations corroborated these findings, consistently showing the most rapid and efficient uptake for CL-U, followed by CL, with free curcumin demonstrating the lowest cellular accumulation (Fig. 8 and Fig. S3).

These results clearly demonstrate that LBP can significantly enhance the cellular uptake of curcumin. We hypothesize that this enhancement may be attributed to two potential mechanisms. First, LBP may inhibit the efflux function of P-glycoprotein (P-gp) on the tumor cell membrane. This is supported by a previous finding that tansy polysaccharide can inhibit P-gp activity in Caco-2 cells [32], which would reduce the efflux of intracellular curcumin and promote its accumulation. Second, similar to reports on jujube polysaccharide and lipopolysaccharides, LBP may interact with the lipid bilayer of the cell membrane, transiently disrupting its ordered structure and increasing membrane permeability or fluidity [33,34]. This membrane-perturbing effect could facilitate the passive diffusion of curcumin molecules into the cells, thereby improving uptake efficiency.

The significantly enhanced cellular uptake of the CL-U, compared to both the CL and free curcumin, can be primarily attributed to the profound impact of ultrasonication on the physicochemical properties of the nanocomplex. We propose that the markedly higher uptake efficiency of CL-U is a direct consequence of its optimized nanostructure, achieved through ultrasonic processing. Firstly, ultrasonication promoted the formation of a more homogeneous and compact core–shell structure, as evidenced by TEM and DLS, with a higher curcumin payload. This increased drug loading directly provides more bioavailable curcumin per complex internalized by the cells. Secondly, the ultrasonication-refined nanocomplex exhibited a more negative zeta potential, which enhances colloidal stability and may favor interactions with the negatively charged cell membrane, potentially facilitating endocytic pathways. Furthermore, the potentiated hydrogen bonding network within CL-U, confirmed by FTIR, likely contributes to its superior stability in the extracellular environment, preventing premature drug release and ensuring that a greater proportion of intact complexes reach and enter the cells. Therefore, the superior cellular uptake performance of CL-U is not merely due to the presence of LBP, but is critically dependent on the structural and stability enhancements imparted by the ultrasonication process. It is worth noting that while the current study focused on elucidating the uptake mechanism in tumor cells, future work will utilize Caco-2 intestinal epithelial models to further investigate the absorption and transport characteristics of CL-U across biological barriers, which will provide critical insights into its oral bioavailability and in vivo applicability.

### 3.8 Evaluation of antioxidant activity of CL-U

**Fig. 9:**
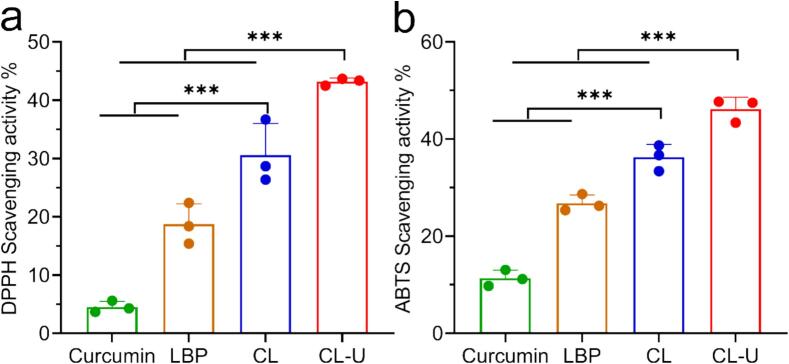
Antioxidant activity of CL-U. a. DPPH. b. ABTS. Data are represented as mean ± s.d. ns, no significance, ***, p < 0.001.

The individual antioxidant properties of curcumin and LBP are well established, contributing to their recognized roles in delaying aging and prevention of chronic diseases [35,36]. Given that CL-U is a complex developed from these two constituents, evaluation of its antioxidant capacity is essential. The radical scavenging activity of CL-U was assessed in vitro using both DPPH and ABTS assays (Fig. 9). Results demonstrated that CL-U exhibited significantly enhanced scavenging rates of 43.1 ± 0.62 % for DPPH and 51.9 ± 5.25 % for ABTS, compared to those of CL (30.6 ± 5.40 % and 37.6 ± 3.77 %, respectively). This improvement in antioxidant efficacy may be attributed to the higher curcumin content in CL-U facilitated by the ultrasonication process. All measurements were conducted at their respective preparation concentrations (CL: 12.87 ± 1.96 mg/mL; CL-U: 12.65 ± 1.32 mg/mL).

### 3.9 Mechanistic insights into CL-U formation

**Fig. 10:**
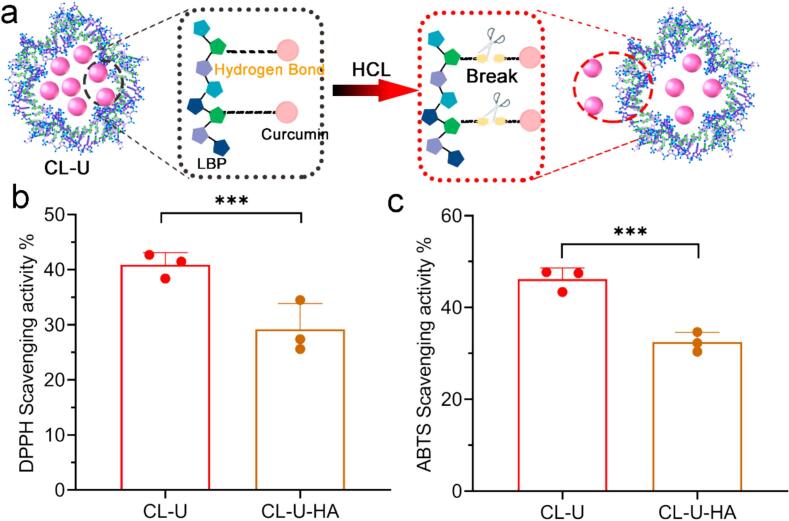
Effect of acid hydrolysis on the antioxidant capacity of the CL-U. (a). Schematic Diagram of acid hydrolysis (b) DPPH (c) ABTS. Data are represented as mean ± s.d. ***, p < 0.001.

To elucidate the intermolecular interactions responsible for the formation of the ultrasonically synthesized CL-U, we conducted acid hydrolysis experiments. The selection of this methodology was based on the established principle that hydrogen bonds are particularly vulnerable to acidic environments, where protonation disrupts these specific non-covalent interactions [37]. Upon treatment with hydrochloric acid, the CL-U solution immediately formed a visible precipitate after vortexing and centrifugation. Quantitative HPLC analysis confirmed a substantial reduction in soluble curcumin content, which decreased to 1.008 ± 0.221 μg in the acid-treated group. This loss of complex integrity directly correlated with a significant diminution of antioxidant efficacy, as measured by both DPPH and ABTS radical scavenging assays (Fig. 10). The concordance of these results provides compelling evidence that hydrogen bonding is a critical intermolecular force responsible for the formation and stability of the CL-U nanocomplex.

## 4 Conclusion

This study establishes an efficient strategy for enhancing the aqueous solubility and bioavailability of curcumin through ultrasonic-assisted complexation with LBP. Systematic optimization revealed that LBP concentration, ultrasonic time, power, and temperature act synergistically to govern the formation of the CL-U. Structural characterization by TEM confirmed the formation of spherical core–shell nanostructures, wherein the hydrophilic LBP shell encapsulates the hydrophobic curcumin core. Most importantly, ultrasonication played a critical role in promoting molecular-level interactions, as evidenced by FTIR spectroscopy showing significantly enhanced hydrogen bonding in the CL-U complex. This optimized architecture led to a 2.23-fold increase in curcumin loading and excellent colloidal stability over five days.

The significantly improved aqueous solubility of curcumin within the CL-U complex directly translated into a marked enhancement in its bioavailability. This was most evident in the dramatically improved cellular uptake efficiency and velocity in 4T1 cells, where CL-U achieved near-complete internalization within one hour—a performance far surpassing both free curcumin and the non-ultrasonicated complex (CL). This suggests that the ultrasonication-refined complex facilitates more efficient cellular delivery. Furthermore, the CL-U complex demonstrated superior antioxidant activity in both DPPH and ABTS assays, a benefit directly correlated with its higher curcumin content and improved dispersibility. The pivotal role of hydrogen bonding in stabilizing the complex was definitively verified through acid hydrolysis, which disrupted these interactions and led to immediate precipitation and loss of bioactivity.

In summary, this work highlights the fundamental value of ultrasonication as a green and efficient sonochemical tool in potentiating the functionality of polysaccharide-based delivery systems. By enabling the construction of a well-defined nanoscale architecture with intensified non-covalent interactions, ultrasonication significantly elevates the performance of LBP as a natural carrier [38]. These findings provide not only a practical methodology for improving the delivery of lipophilic bioactive compounds but also a generalizable strategy for leveraging physical processing to amplify the functional potential of natural polysaccharides in food and pharmaceutical applications [39].

## CRediT authorship contribution statement

**Li-Qiang Zhao:** Writing – original draft, Data curation, Conceptualization. **Zhuo-Qiong Li:** Data curation. **Yue-Fan Liu:** Data curation. **Meng-Ting Jiang:** Data curation. **Ya-Nan Liu:** Formal analysis. **Xin-Lan Zhang:** Formal analysis. **Yu-Jie Sun:** Formal analysis. **Jia-Lun Duan:** Investigation. **Chun-Jie Bao:** Investigation. **Jin-Ao Duan:** Investigation, Funding acquisition.

## Funding

The authors acknowledge financial support from National Natural Science Foundation of China (Grant No. 82405025, U21A20408), Natural Science Foundation of the Jiangsu Higher Education Institutions (Grant No. 23KJB360010, 24KJB360017), Natural Science Foundation of Jiangsu Province (Grant No. BK20230462, BK20240749), Jiangsu Provincial Double-Innovation Doctor Program (JSSCBS20230146, JSSCBS0213).

## Declaration of Competing Interest

The authors declare no interest conflict. They have no known competing financial interests or personal relationships that could have appeared to influence the work reported in this paper.
