# Supplementary material for: Ultrasonic complexation with Lycium barbarum polysaccharide significantly enhances the aqueous solubility and bioavailability of curcumin
Source: Ultrason Sonochem. 2025 Nov 9;123:107673. doi: 10.1016/j.ultsonch.2025.107673 (PMC12651804; doi:10.1016/j.ultsonch.2025.107673)
Supplement: Supplementary Data 1 [file mmc1.docx]

**Ultrasonic complexation with** ***Lycium barbarum* polysaccharide significantly enhances the aqueous solubility and bioavailability of curcumin**

Li-Qiang Zhao^1，†^,Zhuo-Qiong Li^1，†^, Yue-Fan Liu^1，†^, Meng-Ting jiang^1，†^, Ya-Nan Liu^1^, Xin-Lan Zhang^1^, Yu-Jie Sun^1^, Jia-Lun Duan ^1*^, Chun-Jie Bao^1,2*^, Jin-Ao Duan^1*^

^1^ Jiangsu Provincial Key Laboratory of Functional Substances in Traditional Chinese Medicine Formulae and Innovative Drug Discovery, National and Local Collaborative Engineering Center of Chinese Medicinal Resources Industrialization and Formulae Innovative Medicine, Jiangsu Provincial Key Laboratory of Functional Substances in Traditional Chinese Medicine Formulae and Innovative Drug Discovery, Nanjing University of Chinese Medicine, Nanjing 210023, China

^2^ School of Medicine, Nanjing University of Chinese Medicine, Nanjing 210023, China

† These authors contributed equally

* Corresponding authors:

chunjie@njucm.edu.cn (Chun-Jie Bao)

jialun.duan@njucm.edu.cn (Jia-Lun Duan)

dja@njucm.edu.cn (Jin-Ao Duan)


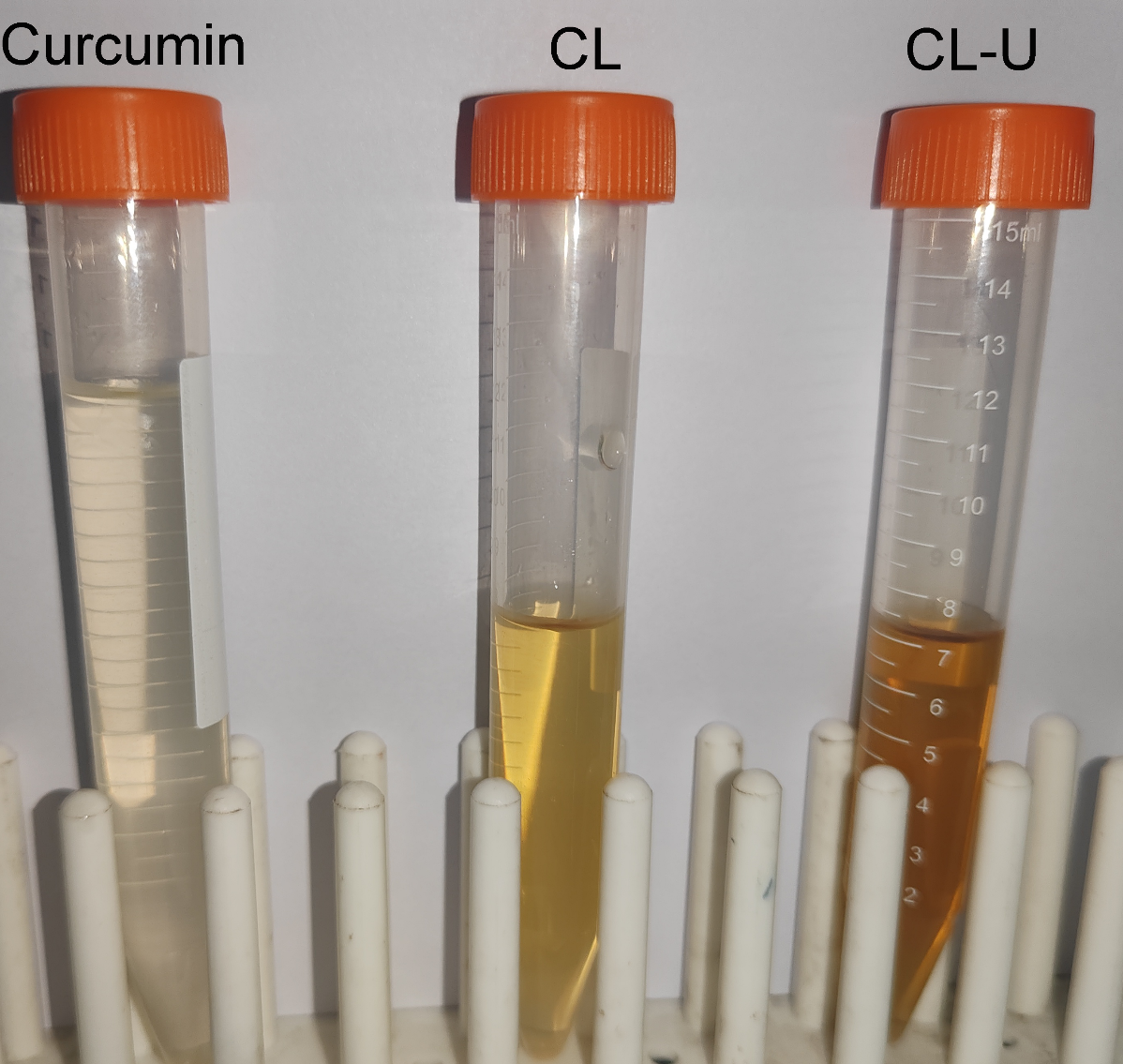


**Fig. S1** **Representative images of curcumin aqueous solution, CL, and CL-U samples.**


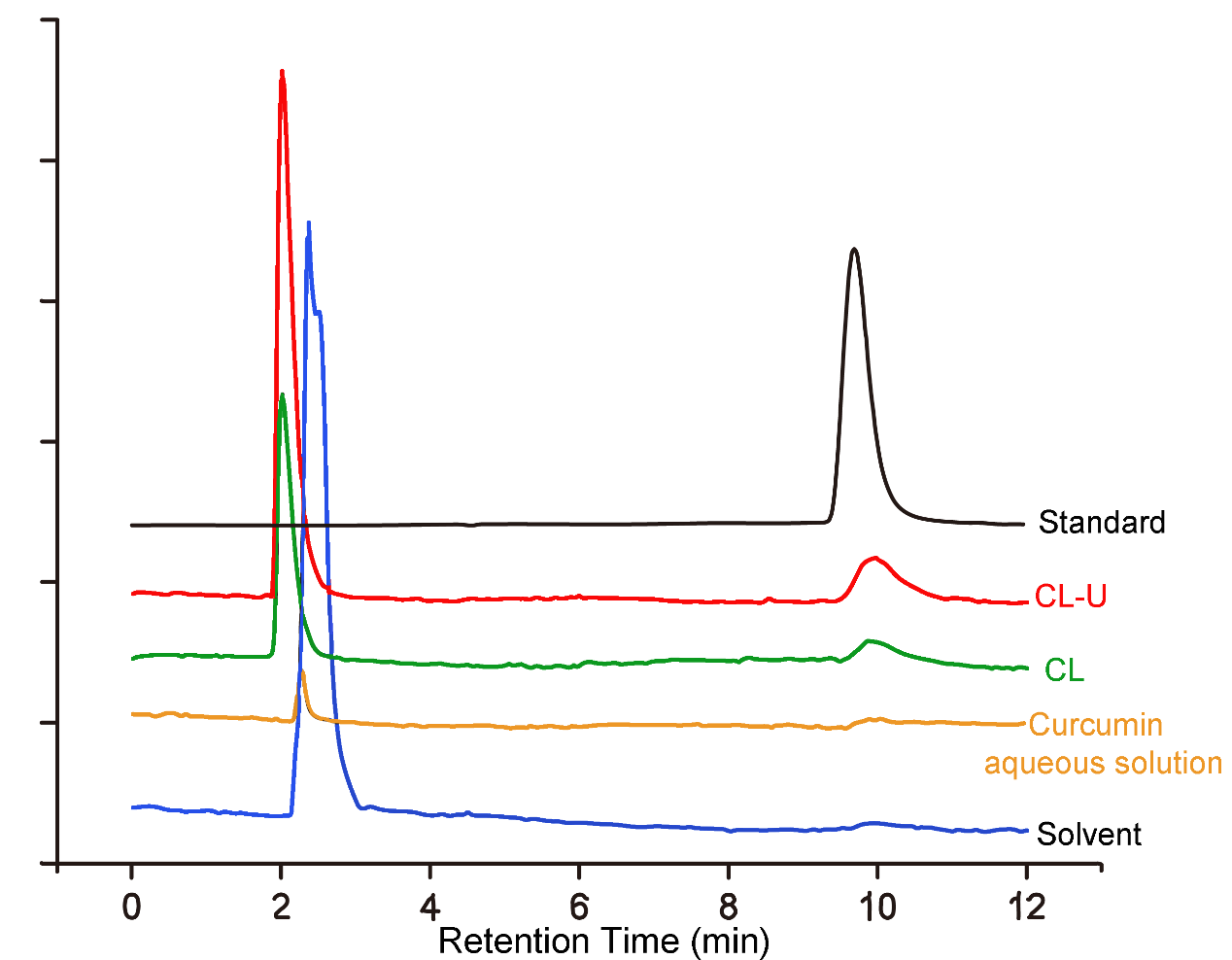


**Fig. S2 HPLC diagram of standard, CL-U, CL, curcumin aqueous solution and solvent.**

**
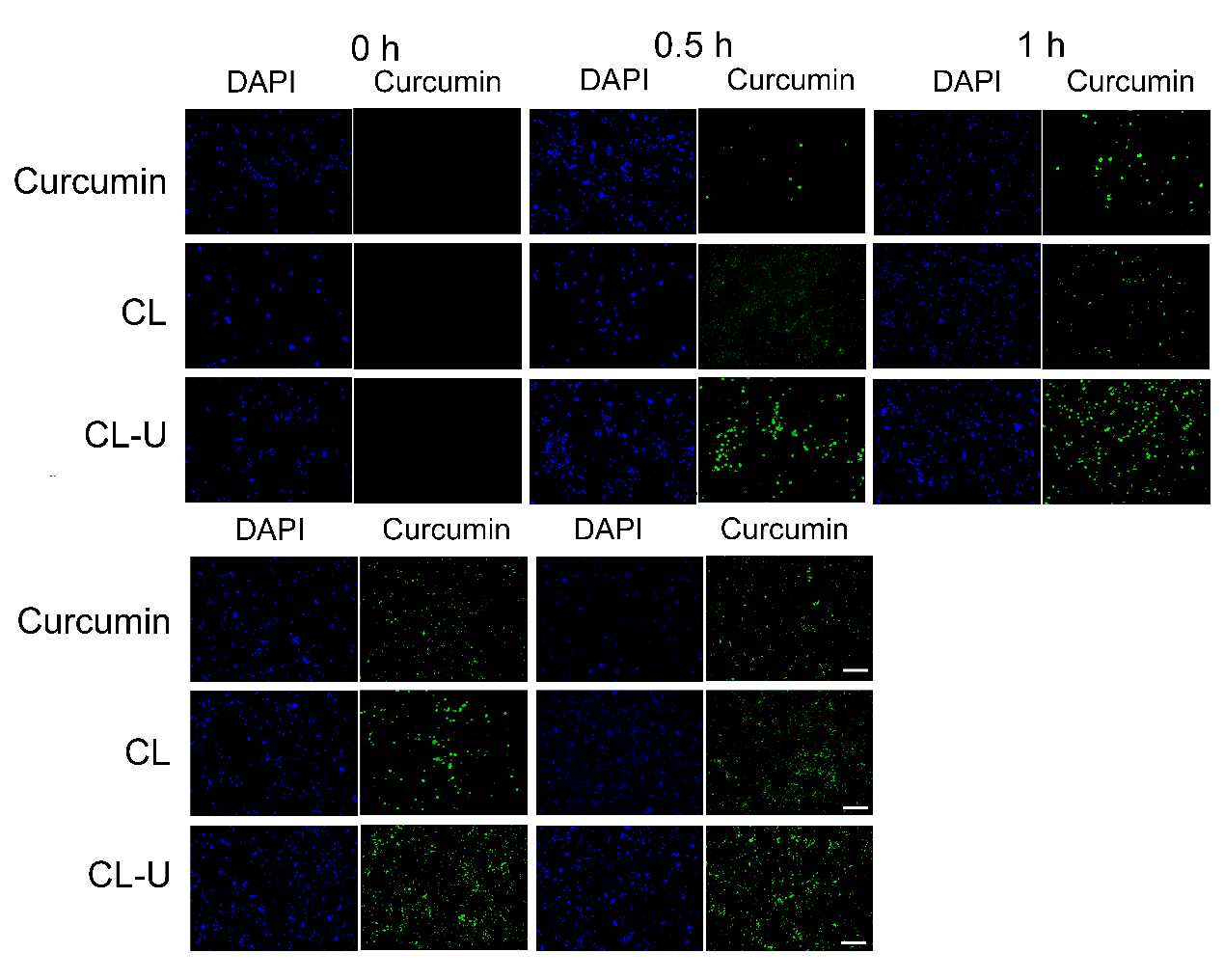
**

**Fig. S3** **Representative images from each group is shown by confocal microscopy.** Scale bars, 200 μm.
